# Multicolor Layer-by-Layer films using weak polyelectrolyte assisted synthesis of silver nanoparticles

**DOI:** 10.1186/1556-276X-8-438

**Published:** 2013-10-22

**Authors:** Pedro Jose Rivero, Javier Goicoechea, Aitor Urrutia, Ignacio Raul Matias, Francisco Javier Arregui

**Affiliations:** 1 Nanostructured Optical Devices Laboratory, Electric and Electronic Engineering Department, Public University of Navarra, Edif.Los Tejos, Campus Arrosadía, 31006, Pamplona, Spain

**Keywords:** Multicolor films, Layer-by-Layer assembly, Silver nanoparticles

## Abstract

In the present study, we show that silver nanoparticles (AgNPs) with different shape, aggregation state and color (violet, green, orange) have been successfully incorporated into polyelectrolyte multilayer thin films using the layer-by-layer (LbL) assembly. In order to obtain colored thin films based on AgNPs is necessary to maintain the aggregation state of the nanoparticles, a non-trivial aspect in which this work is focused on. The use of Poly(acrylic acid, sodium salt) (PAA) as a protective agent of the AgNPs is the key element to preserve the aggregation state and makes possible the presence of similar aggregates (shape and size) within the LbLcolored films. This approach based on electrostatic interactions of the polymeric chains and the immobilization of AgNPs with different shape and size into the thin films opens up a new interesting perspective to fabricate multicolornanocomposites based on AgNPs.

## Background

The synthesis of metal nanoparticles (gold, silver, palladium, copper) and their further incorporation into thin films is of great interest for applications in antibacterial coatings [1,2], catalysis [3,4], chemical sensors [5,6], drug delivery [7,8], electronics [9], photochemistry [10] or photonics [11,12]. The wide variety of synthesis methodologies to obtain the metallic particles provide alternative ways to synthesize the nanoparticles controlling various parameters such as the shape, size, surface functionalization or interparticle distance which affect their final properties. A control of these parameters is a challenging goal, and a large number of reports have been published [13-20]. Among them, the synthesis routes based on the chemical reduction in organic solvents or in which polymers can act simultaneously as a stabilizer and reducer agent to obtain metal nanoparticles have been investigated [21,23]. However, the use of organic media and the synthesis of polydisperse nanoparticles limit their use for some specific applications in where monodisperse nanoparticles are required [24,25].

Alternative procedures for the synthesis of Au or AgNPs are based on the use of water soluble polymers with the aim of achieving size-controlled nanoparticles. Wang and co-workers have obtained AuNPs in aqueous solution in the 1–5 nm size range with the use of poly(methacrylic acid) (PMMA) [26,27]. Keuker-Baumann and co-workers reported a study about the formation of AgNPs with a high control and a characteristic plasmon band at 410 nm is observed using dilute solutions of long-chain sodium polyacrylates (NaPA) by exposing the solutions to UV-radiation [28] in where the coil size of the polymeric chains acts as a collector of silver cations (Ag+). Other researches have investigated the formation of AgNPs and intermediate clusters in polyacrylate aqueous solutions by chemical reduction of Ag + using a reducing agent, gamma radiation or ambient light [29-32]. Very recently, our group has described the synthesis of multicolor silver nanoparticles with a high stability in time, using poly(acrylic acid, sodium salt) (PAA) as a protective agent, in where the AgNPs exhibit localized surface plasmon resonance (LSPR) spectra (colors) as a function of variable protective and reducing agents with a well-defined shape and size [33].

Once the metallic nanoparticles have been synthesized, a further assembly in the form of thin films is required to obtain the desired silver nanoparticle composites. However, this is not always possible because of the need of preserving the aggregation state of the nanoparticles. Several approaches are based on the incorporation of the nanoparticles into a previous polymeric matrix obtained by different thin film techniques, such as sol–gel deposition or electrospinning process [34,35]. In all the cases, the presence of an intense absorption band at 410 nm is indicative of spherical AgNPs with a characteristic yellow coloration. In this work, layer-by-layer (LbL) assembly allows to manipulate and incorporate the nanoparticles into the thin films due to the use of PAA as a protective agent which maintains unaltered the aggregation state of the AgNPs. This technique is based on the alternating deposition of oppositely charged polyelectrolytes in water solution (polycations and polyanions) on substrates where the electrostatic interaction between these two components of different charge is the driving force for the multilayer assembly [36]. Previous works are based on the in situ synthesis of AgNPs in the polyelectrolyte multilayers via counterion exchange and posterior reduction [37-41]. In these cases, this approach is based on the pH-dependent dissociation of weak acids such as PAA as a function of the pH, in where both ionized (carboxylate) and non-ionized (carboxylic) groups are obtained. The presence of the free ionic groups makes possible to bind metal ions via a simple aqueous ion exchange procedure and a posterior chemical reduction step with a reducing agent, leads to obtain the nanoparticles within the thin film. However, Su and co-workers have demonstrated the incorporation of AgNPs with the use of strong polyelectrolytes, such as poly(diallyldimethylammonium chloride) (PDDA) and poly(styrene sulfonate) (PSS), without any further adjustment of the pH [42]. Although the film thickness of the polymeric matrix can be perfectly controlled by the number of layers deposited onto the substrate, a better control over particles size and distribution in the films are not easy to achieve with the in situ chemical reduction and as a result, only yellow coloration is observed. Our hypothesis for obtaining the color is due to a greater degree control over particles (shape and size distribution) in the films with a real need of maintaining the aggregation state.

To overcome this situation, we propose a first stage of synthesis of multicolorAgNPs (violet, green and orange) in aqueous polymeric solution (PAA) with a well-defined shape and size. A second stage is based on the incorporation of these AgNPs into a polyelectrolyte multilayer thin film using the layer-by-layer (LbL) assembly. To our knowledge, this is the first time that a study about the color formation based on AgNPs is investigated in films preserving the original color of the solutions.

## Methods

### Materials

Poly(allylamine hydrochloride) (PAH) (Mw 56,000), Poly(acrylic acid, sodium salt) 35 wt% solution in water (PAA) (Mw 15,000), silver nitrate (>99% titration) and boranedimethylamine complex (DMAB) were purchased from Sigma-Aldrich and used without any further purification.

### Synthesis method of the PAA-capped AgNPs

Multicolor silver nanoparticles have been prepared by adding freshly variable DMAB concentration (0.033, 0.33 and 3.33 mM) to vigorously stirred solution which contained constant PAA (25 mM) and AgNO_3_ concentrations (3.33 mM). This yields a molar ratio between the protective and loading agent ([PAA]/[AgNO_3_] ratio of 7.5:1. The final molar ratios between the reducing and loading agents ([DMAB]/[AgNO_3_] ratio) were 1:100, 1:10 and 1:1. The reduction of silver cations (Ag^+^) and all subsequent experiments were performed at room conditions and stored at room temperature. More details of this procedure can be found in the literature [33].

### Fabrication of the multilayer film

Aqueous solutions of PAH and PAA with a concentration of 25 mM with respect to the repetitive unit were prepared using ultrapure deionized water (18.2 MΩ · cm). The pH was adjusted to 7.5 by the addition of a few drops of NaOH or HCl. The LbL assembly was performed by sequentially exposing the glass slide (substrate) to cationic polyelectrolyte poly(allylamine hydrochloride) (PAH) and anionic polyelectrolyte PAA loaded with the silver nanoparticles previously synthesized (PAA-Ag NPs) with an immersion time of 5 minutes. A rinsing step of 1 minute in deionized water was performed between the two polyelectrolytes baths and a drying step of 30 seconds was performed after each rinsing step. The combination of a cationic monolayer with an anionic monolayer is called bilayer. The LbL process was carried out using a 3-axis cartesian robot from Nadetech Innovations. More details of the LbL assembly can be found elsewhere [35,36,43]. No atmospheric oxidation of the LbL films with AgNPs was observed using this experimental process, showing the long-term stability of the resultant films.

### Characterization

UV-visible spectroscopy (UV–vis) was used to characterize the optical properties of the multicolor silver nanoparticles and the resultant coatings obtained by LbL assembly. Measurements were carried out with a Jasco V-630 spectrophotometer.

Transmission electron microscopy (TEM) was used to determine the morphology (shape and size) of the silver nanoparticles obtained in aqueous solution. This TEM analysis was carried out with a Carl Zeiss Libra 120. Samples for TEM were prepared by dropping and evaporating the solutions onto a collodion-coated copper grid.

Atomic force microscope (AFM) in tapping mode (Innova, Veeco Inc.) has been used in order to show the distribution of the Ag NPs, thickness and roughness of the films obtained by the LbL assembly.

## Results and discussion

**Figure 1:**
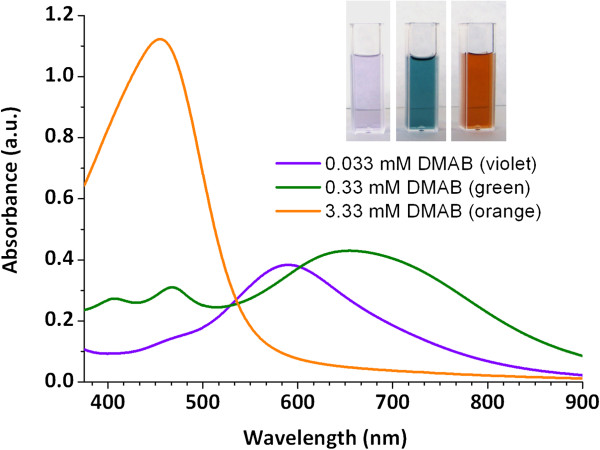
UV–vis spectroscopy of the multicolor silver nanoparticles (violet, green, orange) as a function of DMAB concentration.

In Figure 1, it is possible to appreciate three different colors obtained (violet, green and orange) using PAA as an encapsulating agent (PAA-AgNPs) when DMAB concentration is increased (from 0,033 mM to 3.33 mM). These poly(acrylic acid)-coated nanoparticles are unique in this respect because prior studies using different encapsulating agents to synthesize silver nanoparticles indicate that only an orange coloration is obtained without any color variation. In addition, the resultant PAA-AgNPs dispersions showed an excellent long-term stability since no changes in the position of their absorption bands have been observed after more than one year of storage at room conditions, corroborated by UV–vis spectroscopy.

**Figure 2:**
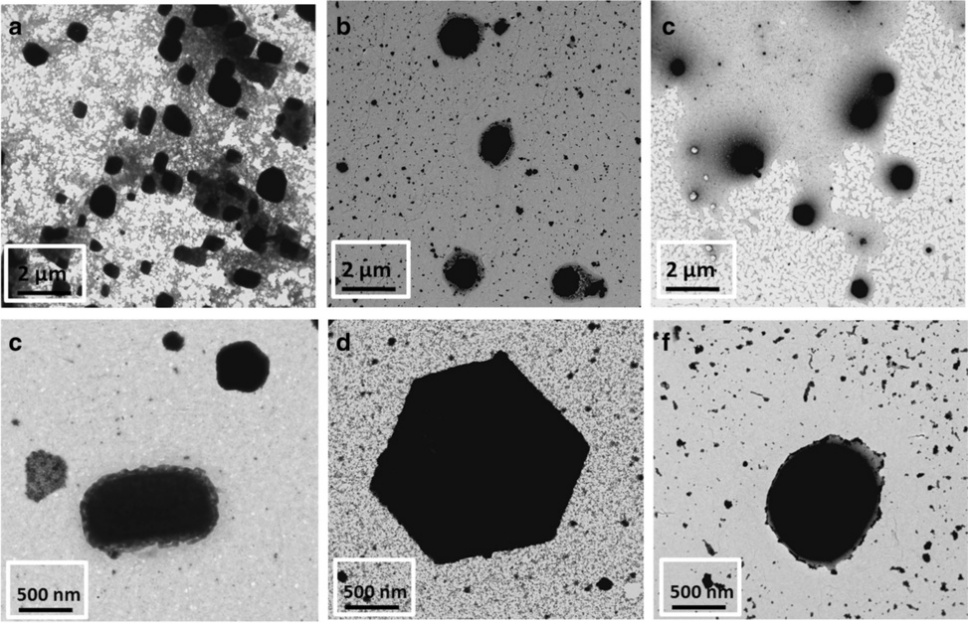
**TEM micrographs of the multicolor silver nanoparticles at different scale (500 nm and 2 μm). (a,d)** rod shape (violet coloration); **(b,e)** hexagonal shape (green coloration); **(c,f)** spherical shape (orange coloration).

Initially, the mixture of 25 mM PAA with AgNO_3_ is colorless (control), but after the addition of 0.033 mM of DMAB, the mixture turns quickly to violet with a plasmonic absorption peak with a maximum centered at 600 nm. When DMAB concentration is increased (0.33 mM), the sample changes from violet to green. The absorption band distribution in the UV–VIS spectrum was altered significantly. The initial absorption band was increased significantly, and it was also shifted toward longer-wavelengths (at 650 nm). Furthermore, a new absorption band was found at 480 nm related with the coexistence of different Ag-NP aggregation states or shapes. Finally, when DMAB concentration is increased to 3.33 mM, the solution turned to orange color and only an intense absorption band around 440 nm was observed, indicating the complete synthesis of spherical silver nanoparticles. The evolution of these absorption bands in two well separated regions (region 1 for the 400–500 nm and region 2 for the 600–700 nm) has been discussed in previous works [33]. These changes in the UV–vis spectra (colors) are related to changes in the shape, size and aggregation state of the AgNPs. In order to corroborate this hypothesis, TEM analysis of the different samples (PAA-AgNPs) were performed (see Figure 2).

According to the results observed in Figures 1 and 2, when DMAB concentration added in the reaction mixture is low, violet coloration ([DMAB]/[AgNO_3_] = 0.01) or green coloration ([DMAB]/[AgNO_3_] = 0.1) is observed with a typical long-wavelength absorption band (600–700 nm) and a new absorption band at 480 nm appears for green coloration, which corresponds to complexes of small positively charged metal clusters and polymer ligands of the polyacrylate anions (PAA) [44-46]. It has been also found that AgNPs with a specific shape and size (TEM micrographs), nanorods of different size (from 100 to 500 nm) are synthesized for violet coloration. Additionally, clusters with a hexagonal shape (from 0.5-1 μm) mixed with spherical particles of nanometricsize are found for green coloration. However, when DMAB concentration is increased ([DMAB]/[AgNO3] = 1), orange coloration with an intense absorption band at 440 nm is observed, which is indicative of a total reduction of the silver cations and the corresponding synthesis of spherical nanoparticles with variable size. These results corroborate that the excess of free Ag^+^cations immobilized into the polyelectrolyte chains of the PAA respect to the reducing agent, plays a key role in the synthesis process, yielding different nanoparticle size distributions and aggregation states. It is important to remark that changes in the plasmonic absorption bands (resultant color) basically depend on the relationship between the aggregation state of the nanoparticles (even in the cluster formation) and the final shape/size of the resultant nanoparticles. A control of all these parameters is the key to understand the color formation in the films.

**Figure 3:**
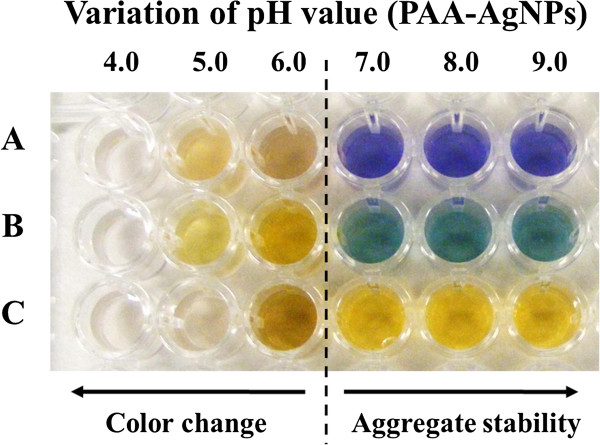
Variation of the multicolor silver nanoparticles (PAA-AgNPs) as a function of the pH value for violet (A), green (B) and orange coloration (C).

The next step is to incorporate the previously synthesized colored AgNPs in a polyelectrolyte multilayer film using the layer-by-layer (LbL) assembly. The main goal is to get a coating with the similar coloration that the initial colored solution of PAA-AgNPs (violet, green and orange). Therefore, it is necessary to maintain the aggregation state of the nanoparticles into the thin film. Then, the multilayer assembly of both cationic polyelectrolyte poly(allylamine hydrochloride) (PAH) and anionic polyelectrolyte PAA loaded with the AgNPs previously synthesized (colored PAA-Ag NPs) depends on the degree of ionization of the polymers and their charge density which is perfectly controlled with a suitable adjustment of the pH [47,48]. An important consideration of this work is that the deposition of PAH and PAA-AgNPs is at the same pH (7.5) because PAA at this pH or higher pH values plays a key role in order to preserve the aggregation state of the nanoparticles during the synthesis process (Figure 3) with a perfect control of the resultant color without any further precipitation. When the pH of the dipping solutions (PAA-AgNPs) is lowered below 7.0, a change of the coloration is observed in all the experiments which it is indicative of a loss of the aggregation state of the PAA-AgNPs with an increase in opalescence and a further precipitation with a complete loss of color (transparent solutions) at low pH values (pH 4.0 or lower).

**Figure 4:**
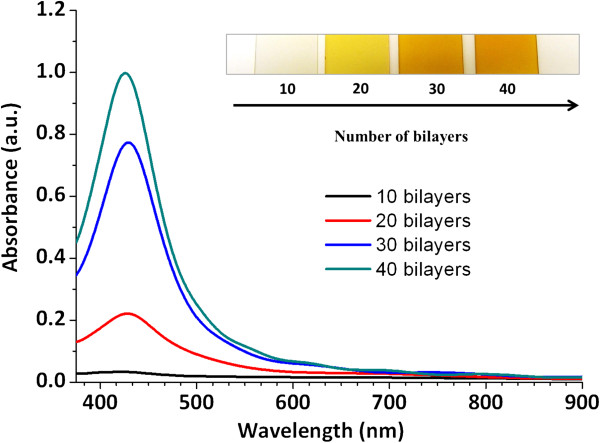
UV–vis spectroscopy of the orange multilayer films for different number of bilayers (10, 20, 30 and 40) and photographs of the coatings.

Due to these changes concerning to the color as a function of the pH dipping solutions, the reason of choosing pH 7.5 for both PAH and PAA-AgNPs is the base to obtain the multicolor films. In addition, the fundamental element to obtain the multilayer buildup is the presence of ionized groups of these weak polyelectrolytes, which are responsible for the electrostatic assembly and the spatial control of the previously silver nanoparticles distribution (colored PAA-AgNPs) in the multilayer film when the number of bilayers is increased. In Figure 4, a detail of the evolution of the absorption peaks (UV–vis spectroscopy) and the corresponding color formation during the LbL fabrication process for both PAH and PAA-AgNPs (orange coloration) is shown as a function of the number of bilayers added to the corresponding films.

From the results of Figure 4, it can be said that a successful deposition of orange colored films was obtained. A LSPR absorption peak centred at 440 nm grows as a function of the number of bilayers deposited onto glass slides via LbL assembly (10, 20, 30 and 40 bilayers, respectively). The intensity increase of the absorption band at 440 nm or the orange coloration of the films, is the result of an incorporation of spherical AgNPs in the multilayer assembly.

**Figure 5:**
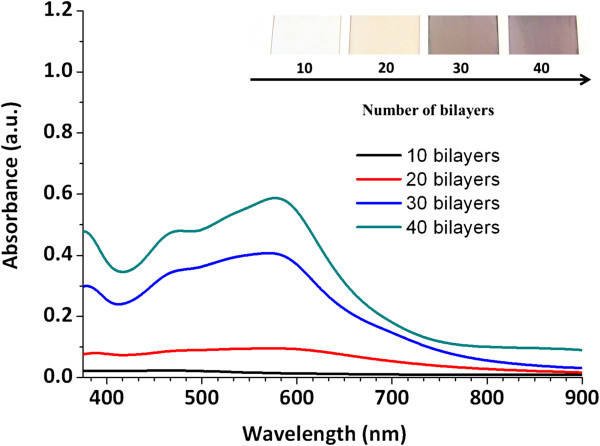
UV–vis spectroscopy of the violet multilayer films for different number of bilayers (10, 20, 30 and 40) and photographs of the coatings.

As it has been previously commented, the aim of this manuscript is to get thin films with the same coloration that the initial PAA-AgNPs solution. The next step will be to incorporate the violet silver nanoparticles in the LbLbuildup. In Figure 5, a study of the evolution of the absorption bands corresponding to both PAH and PAA-AgNPs (violet) during the LbL fabrication process is studied at the same number of bilayers.

According to the results, an increase of the absorption peak from 10 bilayers to 40 bilayers at a specific wavelength position is observed. The location of this absorption band, which is higher in intensity when the thickness of the coating is increased, maintains the same position that initial synthesized violet silver nanoparticles (PAA-AgNPs) at 600 nm (see Figure 1). In view of these results, UV–vis spectra reveal identical absorption peaks for both LbL fabrication process and the synthesized PAA-AgNPs (violet solution), which it means that silver nanoparticles with a specific shape (mostly rods) have been successfully incorporated in the multilayer assembly.

**Figure 6:**
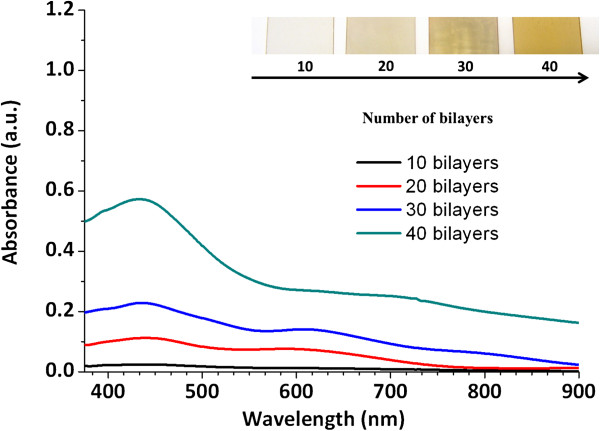
UV–vis spectroscopy of the green multilayer films for different number of bilayers (10, 20, 30 and 40) and photographs of the coatings.

In Figure 6, the evolution of the absorption bands corresponding to the coating of PAH and PAA-AgNPs (green) during LbL fabrication process is shown. UV–vis spectra of the resulting coatings at different number of bilayers confirm the existence of two absorption peaks during the multilayer assembly, one at 640 nm typical of green AgNPs which is lower in intensity and the other one, higher in intensity at 440 nm. For this case, it is possible to appreciate a difference in the UV–vis spectra between the LbL multilayer assembly and the previously green colored PAA-AgNPs (see Figure 1). In the opinion of the authors, the presence of a higher and broader absorption band at 440 nm is due to an agglomeration and higher number of the AgNPs inside of the thin film and the presence of AgNPs with different shape (not only hexagonal shape). This approach is corroborated by the final coloration of the resultant coatings in where a light orange coloration instead of clearly green coloration is observed. A possible reason of this spectral change (color) in comparison with previously PAA-AgNPs could be associated to the reduction of the metal clusters with a partial positive charge by the amine groups [49,50] of the PAH during the LbL assembly. However, this hypothesis has not been observed for the violet coloration (Figure 5) when the number of bilayers onto glass slides was continuously increased, so we can conclude that a reduction by the amine groups of PAH and a further in situ generation of the spherical AgNPs is not observed. According to the results, the presence of the absorption band at 440 nm is associated to the incorporation of AgNPs with less size (mostly spherical nanoparticles) during the fabrication process (observed by TEM images), whereas the absorption band at 480 nm is lower in intensity because of a more difficult incorporation of higher size particles (metal clusters with hexagonal shape) in the multilayer films for a total number of 40 bilayers. As conclusion, we can remark that a selective absorption process is observed and as result, it is the partial orange coloration of the resultant films due to a higher presence of spherical AgNPs in comparison with hexagonal clusters.

**Figure 7:**
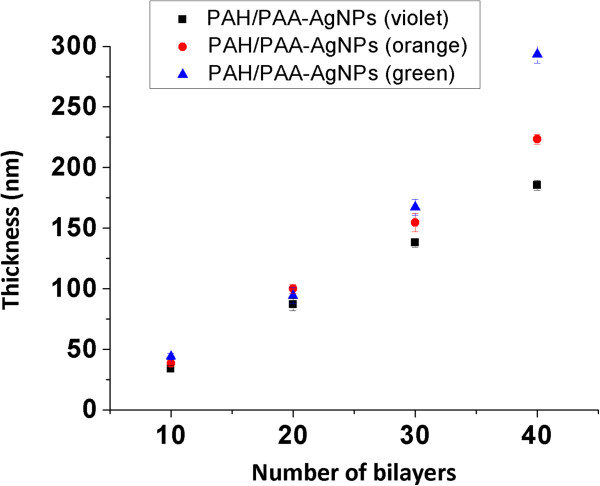
Evolution of thickness of the PAH/PAA-AgNPs multilayer assemblies (violet, green, orange) for different number of bilayers.

In order to understand the incorporation of the multicolorAgNPs inside the LbL assembly, the position of the absorption bands with their corresponding intensities and the aspect in coloration of the final films have been analyzed. However, to create a template of well-defined coloration, the thickness of the resulting films to incorporate the AgNPs plays a key role, which is perfectly controlled by two factors, the pH value of the polyelectrolyte solutions (PAH and PAA-AgNPs) and the number of bilayers deposited onto glass slides [47,48]. When the pH of the dipping solutions is 7.5, both PAH and PAA-AgNPs are adsorbed as fully charged polyelectrolytes and very thin films are obtained. For a total of 40 bilayers, the average thickness is varied from 185 nm (PAH/PAA-AgNPs violet coating), 223 nm (PAH/PAA-AgNPs orange coating) to 293 nm (PAH/PAA-AgNPs green coating). In Figure 7, the evolution of the thickness for different number of bilayers (10, 20, 30 and 40, respectively) with their error bars in this pH regime (7.5) is shown. According to these thickness results, it is possible to appreciate that PAH/PAA-AgNPs with a light orange coloration instead of clearly green coloration is due to the higher incorporation of AgNPs with nanometric spherical size instead of metal clusters into the film for a coating of 40 bilayers.

Obviously, in all the cases of study, the thickness and the resultant color formation depends basically on surface charge of both ionized PAH/PAA polymeric chains, the number of bilayers deposited, the number of the AgNPs incorporated and the distribution of them with a specific shape during the fabrication process. In order to show the aspect of the thin films after LbL fabrication process, AFM images of 40 bilayers [PAH/PAA-AgNPs] at pH 7.5 reveal that the morphologies of the thin films were homogeneous, very slight porous surfaces with an average roughness (rms) of 12.9 nm (violet coloration), 16.7 nm (green coloration) and 18.6 nm (orange coloration). In all the cases, the polymeric chains of the weak polyelectrolytes (PAH and PAA) are predominant in the outer surface and the AgNPs are embedded inside the polymeric films. In order to show the presence of these AgNPs in the LbL assembly, a thermal treatment of the films was necessary with the idea of evaporating the polymeric chains (PAH and PAA, respectively) and so, the contribution of the AgNPs can be appreciated when the fabrication process is performed.

**Figure 8:**
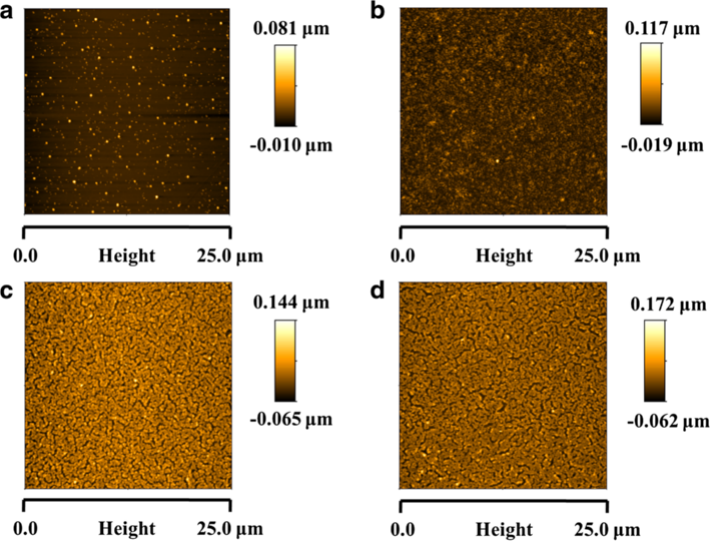
AFM images (25x25 μm) of PAH/PAA-AgNPs (violet coloration) after a thermal treatment as a function of number of bilayers (a) 10 bilayers; (b) 20 bilayers; (c) 30 bilayers and (d) 40 bilayers.

In Figure 8, AFM images corresponding to 10, 20, 30 and 40 bilayers of PAH/PAA-AgNPs (violet coloration) after a thermal treatment of 450°C are shown. In all the images, the only presence of AgNPs is observed and a complete change in the morphology is observed for all the films when the number of bilayers was increased. Initially, when the coating has 10 bilayers it is possible to appreciate well-separated AgNPs with a very low roughness of 5.8 nm. However, when the number of bilayers is increased, the roughness is changing from 10.2 nm (20 bilayers) to 23.9 nm (30 bilayers) and 28.7 nm (40 bilayers). It is important to remark that after a thermal treatment, the total evaporation of the polymeric chains induces an agglomeration of the AgNPs without preserving their distribution along the films. This aspect is corroborated due to a color change from violet to orange in the resultant films.

**Figure 9:**
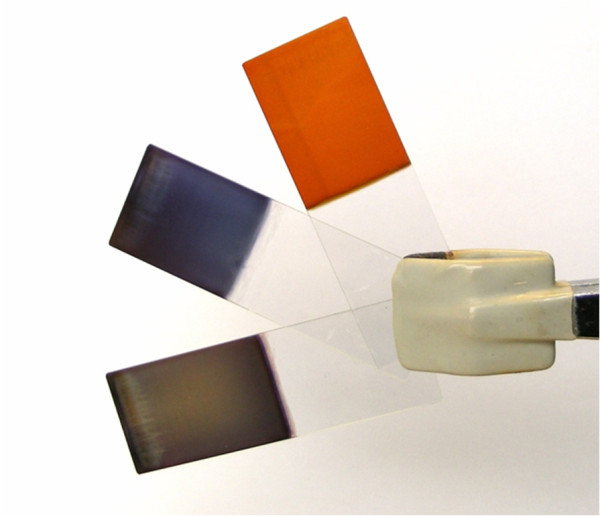
Final aspect of the PAH/PAA-AgNPs multilayer assembly (violet, green, orange coloration) for a total number of 80 bilayers.

In other words, the fact that a higher number of bilayers during the LbL fabrication process, and consequently, a higher thickness of the resultant films, promote a better definition of the color, mostly in the green coloration (see Figure 9) because of a better entrapment of both initial clusters (hexagons with higher size) and nanometric spherical AgNPs in the multilayer assembly. Additionally, new PAH/PAA-AgNPs coatings of 80 bilayers at pH 7.5 have been fabricated in order to show clearly the final coloration onto the glass slides as a function of the initial synthesized multicolor silver nanoparticles (PAA-AgNPs).

**Figure 10:**
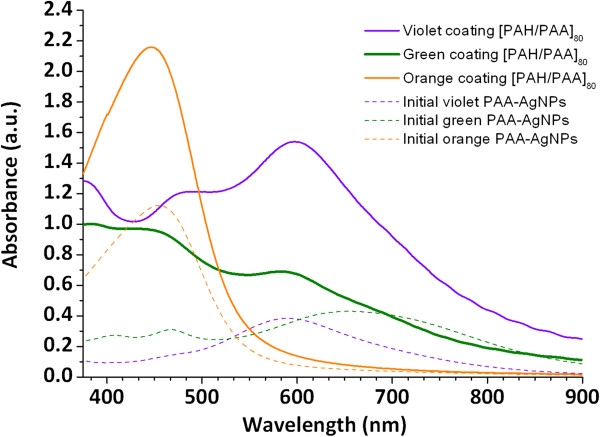
UV–vis spectra of the multilayer thin films of 80 bilayers PAH/PAA-AgNPs (violet, green and orange coloration) in comparison with initial colored PAA-AgNPs solutions.

Figure 10 shows the UV–vis spectra of the samples prepared with this thickness (80 bilayers) and the spectra reveal that the position of the absorption bands is the same than previous spectra (Figures 3, 4 and 5) but with a considerable increase in intensity of the absorption peaks due to a higher number of the metallic silver nanoparticles that have been incorporated into the multilayer film. Therefore, when the thickness is increased, it is possible to corroborate the presence of the same aggregates species or AgNPs than the original colloidal solutions. In other words, when the thickness is increased, the final coloration of the resultant films (violet, green or orange) is similar than the color of the original colloidal PAA-AgNPs solutions. These results of coloration as a function of number bilayers indicate that a higher thickness leads to a better incorporation of higher size aggregates (clusters) in the resultant films. This is the first time that a study about colored AgNPs synthesis and their incorporation in multicolor films (violet, green or orange) is investigated using the LbL assembly. These multicolor LbL films can be used for optical fiber sensor applications [41]. The retention of the color of the Ag-colloidal dispersion in the LbL films makes possible the fabrication of optical fiber sensors with optical responses related to their specific LSPR absorption bands. In such case, different optical fiber sensor signals could be multiplexed into a single optical fiber enabling multipoint measurement.

## Conclusions

In this work, highly stable coloredAgNPs were synthesized using a water-based synthesis route using PAA as capping agent. The weak polyelectrolyte nature of the PAA and the excess of Ag + cations respect to the concentration of reducing agent (DMAB) make possible to achieve nanoparticles with different sizes, shapes and aggregation states. This yields different coloredAgNPs dispersions (violet, green and orange). Such AgNPs have been successfully incorporated into LbL thin films in where the adsorption process was carried out that the AgNPs and aggregates (clusters) within the film are maintained, and thus the coloration of the films is also kept. In order to obtain the proper coloration of the thin film, a study about the influence of the number of PAH/PAA-AgNPs bilayers added (10, 20, 30, 40 and 80, respectively), the position of the absorption bands (UV–vis spectra) and the pH value of the weak polyelectrolytes solutions have been performed. A pH value of 7.5 or higher value of the PAA-AgNPs solution is the key to preserve the aggregation state of the AgNPs without any further precipitation or loss of coloration. A better definition of the coloration in the films is observed when a higher number of bilayers (thickness) are added during the LbL assembly (mostly in green color) because of a better entrapment of both initial clusters and nanometric spherical nanoparticles. This is indicative of a higher number of AgNPs or aggregates of specific shape and size that are incorporated into the multilayer film. In addition, AFM images reveal a low roughness of the resultant colored films which drastically changes with a thermal treatment due a total evaporation of the polymeric chains (PAH and PAA), making possible to appreciate the number of AgNPs incorporated as a function of bilayers added. To our knowledge, this is the first time that colored PAA-AgNPs of different sizes and shapes are synthesized and incorporated later in LbL assemblies preserving the original color of the solutions.

## Abbreviations

PAA: Poly(acrylic acid sodium salt); PAH: Poly(allylamine hydrochloride) (PAH); AgNO3: silver nitrate; DMAB: dimethylaminoborane; LbL: layer-by-layer; LSPR: Localized Surface Plasmon Resonance; AgNPs: silver nanoparticles; TEM: Transmission Electron Microscopy.

## Competing interests

The authors declare that they have no competing interests.

## Authors’ contributions

PJR carried out the main part of the experimental work, and carried out the syntehsis process of the coatings. He participated in the design of the study and in the draft of the manuscript. JG participated in the experimental work, carried out the AFM measurements and contributed with the draft of the manuscript. AU participated in the experimental work and carried out the UV–vis spectra. IRM participated in the design of the study and helped to draft the manuscript. FJA participated in the design of the study and helped to draft the manuscript. All authors read and approved the final manuscript.
